# Disruptive Model That Explains for the Long-Lived Triplet States Observed for 2-Thiocytosine upon UVA Radiation

**DOI:** 10.1021/acsomega.3c09471

**Published:** 2024-03-08

**Authors:** Jorge Baños, Alejandro Avilés, Fernando Colmenares

**Affiliations:** Departamento de Física y Química Teórica, Facultad de Química, Universidad Nacional Autónoma de México, CDMX 04510, Mexico

## Abstract

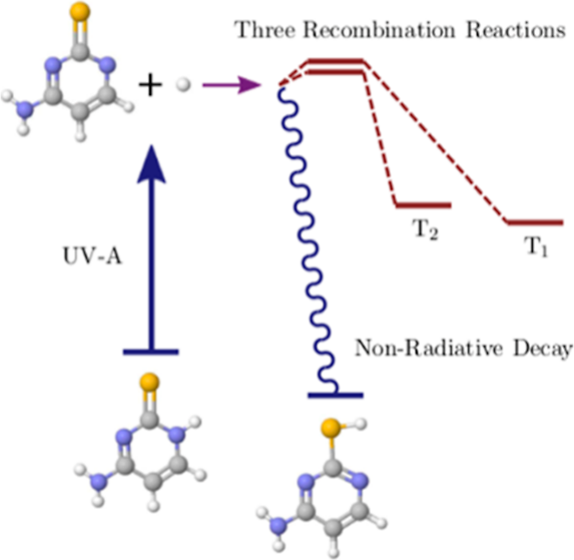

The possible role of radical species in the formation of the long-lived triplet states observed for 2-thiocytosine upon UV irradiation was theoretically investigated. It is predicted that the radical fragments arising from the homolytic rupture of the NH group of the thiobase can be yielded upon ultraviolet-A radiation. Recombination of the radicals through the most favorable singlet channel yields the lowest-lying tautomer of the 2-thiocytosine (the amino-thiol form) through a barrierless pathway. The rebounding of the radical fragments along the triplet channels that emerge from the attack of the hydrogen to the nitrogen atoms next to the C–S bond leads to stable structures for the amino-thion-N_1_H and amino-thion-N_3_H tautomers. These results allow for the rationalization of the near-unity triplet yields observed when this pure light-atom organic molecule is exposed to UV irradiation, without invoking intersystem crossings between the electronic states of different spin-multiplicities. A similar study for cytosine showed that the energy required to induce the homolytic breaking of the N–H bond of the nucleobase is not attainable under UVA radiation. This result is consistent with the experimental fact that no triplet states are observed when this molecule is exposed to that light.

## 1 Introduction

It is known that most of the thio-substituted nucleobases (thiobases), those nucleobase derivatives in which the oxygen atom of a carbonyl group is substituted by a sulfur atom, produce long-lived excited triplet electronic states in near-unity yields upon UVA irradiation.^1^ This has been the subject of many investigations, both experimental and theoretical, due, among others, to the potential use of this kind of noncanonical bases in photodynamic therapy applications as photosensitizers.^2−4^ Interestingly, photoexcitation of the canonical nucleobases with UVA radiation leads in all the cases to a fast nonradiative decay to the singlet ground state, without the formation of excited triplet states.^5−10^ However, triplet structures have been observed for some of them at higher energies (corresponding to the UVB/UVC regions).^11,12^

Different groups have explored theoretically the mechanisms that connect the singlet ground state of the sulfur-substituted nucleobases with the long-lived excited triplet states observed upon UVA radiation.^2,12−20^ In all cases, the proposed mechanisms involve intersystem crossings (ISCs) between the potential energy surfaces belonging to excited singlet and triplet electronic states of the thiobases. The drawback of this kind of description is that the spin–orbit effects that could favor the interaction between electronic states of different spin multiplicities are expected to be significant in molecules containing heavy atoms but not in those composed only by light atoms. For instance, the spin–orbit coupling (SOC) values for systems containing massive atoms can be observed above 500 cm^–1^, whereas for those interactions involving only nonheavy atoms, SOC values usually range between 1 and 100 cm^–1^.^21^ According to the Landau–Zener theory, a low probability for a triplet–singlet surface hopping in pure light-atom molecules should be expected, as the hopping depends on the square of the SOC constant.^22,23^ Particularly, it is unlikely that the hopping probabilities between the excited electronic states of the sulfur-substituted nucleobases will be high enough to explain the near-unity triplet yields observed for them upon UV irradiation. Likewise, the ISC rate from a singlet to a neighbor triplet state estimated through the semiclassical Marcus theory also depends on the squared value of the SOC matrix element *V*_SOC_.^24^
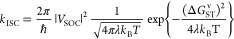
1(In this equation, λ is the reorganization energy, Δ*G*_ST_^v^ represents the vertical free Gibbs energy variation, and *k*_B_ and ℏ are the Boltzmann and the reduced Planck constants, respectively.) Thus, the singlet–triplet conversion through an ISC could hardly account for the ultrafast population of long-lived triplet states observed when the thiobases are exposed to UV irradiation.^1^

In previous reports, we have used a two-step radical reaction scheme to rationalize the shift observed in the spin multiplicity between the reactants and products in reactions leading to the activation of small molecules by transition metal atoms or complexes.^25−30^ According to this scheme, the first reaction evolves to the radical species obtained by hydrogen-atom abstraction from the molecule that is being activated by the metal-containing system (although this abstraction reaction can involve an atom different from hydrogen or even a small molecular moiety).^26,28^ Once the radical species are yielded, they can recombine in a second reaction. For the rebounding of the radicals, in those contributions, we focused our attention in the two lowest-lying radical asymptotes whose electronic configurations differ only in the spin (up or down) of the unpaired electron in the nonmetallic fragment. As those asymptotes are degenerate, recombination of the radicals can take place along two multiplicity channels (a detailed description of the recombination channels for 2-thiocytosine is given below). This reaction scheme allows one to describe the shift in the spin-multiplicity between the reactants and products without invoking ISCs between the electronic states of different spin-multiplicities.

In this paper, we have used a similar scheme to rationalize the fast and near-unity yield of long-lived triplet electronic states observed for the 2-thiocytosine upon UVA irradiation.^20^ As discussed later, the proposed scheme also explains the lack of triplet states when the corresponding canonical base (cytosine) is irradiated with this light.

## 2 Results and Discussion

### 2.1 2-Thiocytosine

**Figure 1:**
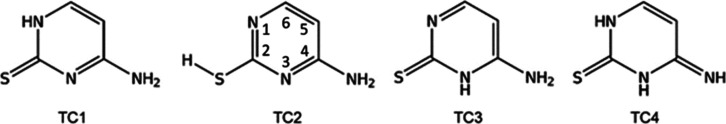
Structures for the lowest-lying singlet tautomers of the 2-thiocytosine: the amino-thion-N_1_H (TC1), amino-thiol (TC2), amino-thion-N_3_H (TC3), and imino-thion (TC4) forms. For reference, the pyrimidine atom-numbering scheme was used in TC2.

First, we explored the possibility that the radical species arising by hydrogen abstraction from the NH and NH_2_ groups of this molecule could be formed by excitation at the UVA and UVB wavelengths employed to record the transient absorption spectra reported in ref (20) (321 and 308 nm, respectively). In Figure 1, structures for the different tautomers of 2-thiocytosine are shown. In the gas phase, TC2 is the most stable tautomer.

According to the data provided in Table 1, the energy of the radical fragments that arise by abstraction of the hydrogen atom from the amino group is 4.20 eV above the ground-state reference, whereas the energy of the radical species obtained by expelling the hydrogen atom from the SH group of TC2 is 3.43 eV. The last energy is below the values of the energies supplied by the UVA and UVB radiations used by Mai et al. to obtain the femtosecond transient absorption spectra for this molecule (3.89 and 4.05 eV, respectively).^20^

**Table 1:** Energies for the Lowest-Lying Singlet Tautomers of 2-Thiocytosine [the Amino-thion-N1H (TC1), Aminothiol (TC2), Amino-thion-N3H (TC3), and Iminothion (TC4) Forms] and Energies for the Singlet and Triplet Radical Fragments Obtained by Hydrogen Abstraction of the NH_2_ and NH Groups of 2-Thiocytosine^a^

	CASPT2 energy (au)	CASSCF-ZPE (au)	CASPT2 + ZPE (au)	relative energy (eV)	relative energy (kcal/mol)
^(T)^TC1_NH__^•^_+ H^•^	–716.287852	0.087516	–716.200336	4.22	97.4
^(S)^TC1_NH__^•^_+ H^•^	–716.288574	0.087516	–716.201058	4.20	97.0
^(T)^TC2^•^ + H^•^	–716.316946	0.088322	–716.228624	3.45	79.7
^(S)^TC2^•^ + H^•^	–716.317692	0.088322	–716.229370	3.43	79.2
TC3	–716.438030	0.102006	–716.336024	0.53	12.3
TC4	–716.447750	0.102496	–716.345254	0.28	6.5
TC1	–716.449542	0.102291	–716.347251	0.22	5.2
TC2	–716.453817	0.098170	–716.355647	0	0

a Energies in columns 5 and 6 are relative to the zero-point energy (ZPE) level of the TC2 tautomer (the most stable form of the molecule in the gas phase). Small variation between the energy values calculated for the singlet and triplet electronic states of each of the investigated radical asymptotes arises from the limited space used to span their corresponding multiconfigurational functions.

**Figure 2:**
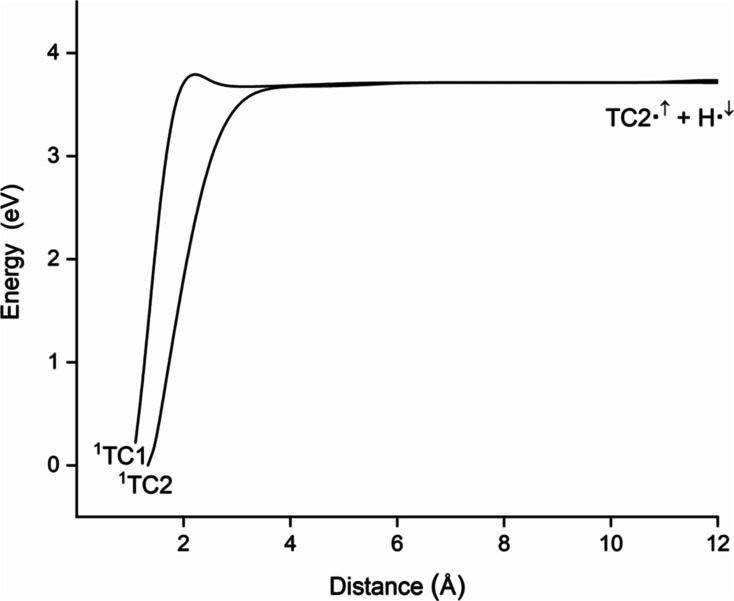
CASPT2 potential energy curves for the fragmentation of the most stable tautomers of 2-thiocytosine into radical fragments (see Table 1).

In Figure 2 are shown the potential energy curves emerging from the two lowest-lying electronic states of 2-thiocytosine to yield the radical fragments TC2^•^ + H^•^ (as TC2^•^ and TC1^•^ correspond to the same doublet structure, both curves evolve to the same radical asymptote). The pathway emerging from the ground state of the thiobase reaches the radical species without energy barriers (i.e., the energy that must be provided to attain the radical fragments is just the energy at which the radical species lie).

The pathway joining tautomer TC1^•^ with the radical species exhibits an energy barrier lying around 0.29 eV above the radical asymptote (this barrier arises from attractive interactions between the hydrogen and sulfur atoms). Importantly, both pathways lie entirely below the radiation energy. This suggests that the radical fragments can be reached from the two tautomers upon the UVA radiation employed to record the transient absorption spectra reported in ref (20). In fact, the energy of those radical fragments differs only by 0.06 eV from the value of 3.49 eV reported in ref (20) for the maximum detected in the UV range of the transient absorption spectra.^20^ Thus, this maximum might be related to the energy required to induce the homolytic rupture in the S–H group of 2-thiocytosine. This has encouraged us to explore the possibility of using the two-step radical reaction scheme mentioned in the preceding section to rationalize the formation of long-lived excited triplet states observed for 2-thiocytosine after UV absorption.

For this, it is first assumed that the singlet state of the radical fragments TC2^•^ + H^•^ is attained by UV excitation. Once these radical species are yielded, they can recombine themselves in a second reaction. For the rebounding reaction, we considered the degenerate lowest-lying singlet and triplet radical asymptotes TC2^•^ + H^•^ whose electronic configurations vary only in the spin up or down of the unpaired electron located in the hydrogen atom (Table 2).

**Table 2:** CASSCF Main Molecular Contributions for the Singlet and Triplet Electronic States of Radical Fragments TC2^•^ + H^•^

	CASSCF coefficient	outermost valence configuration^a^				
^1^A	0.969	↑↓	↑↓	↑↓	↑	↓
^3^A	0.969	↑↓	↑↓	↑↓	↑	↑
		(*p*_*z*_)_S_	Σ – *p*_*z* (C2,N1,N3)_	*p*_*z*(N1) –_ *p*_*z*(N3)_	(*p*_*x*_ – *p*_*y*_)_s_	*s*_H_

a For simplicity, contributions to the molecular orbitals are shown only schematically. In column 4, C2 denotes the carbon atom bonded to sulfur in the fragment TC2^•^. In columns 4 and 5, N1 and N2 represent the nitrogen atoms next to the CS bond (see Figure 1).

Upon UV irradiation, it is equally probable that the singlet radical species will be yielded as TC2^•↑^ + H^•↓^ or TC2^•↓^ + H^•↑^. Thus, recombination of the radical fragments can take place through both spin-multiplicity channels.

**Figure 3:**
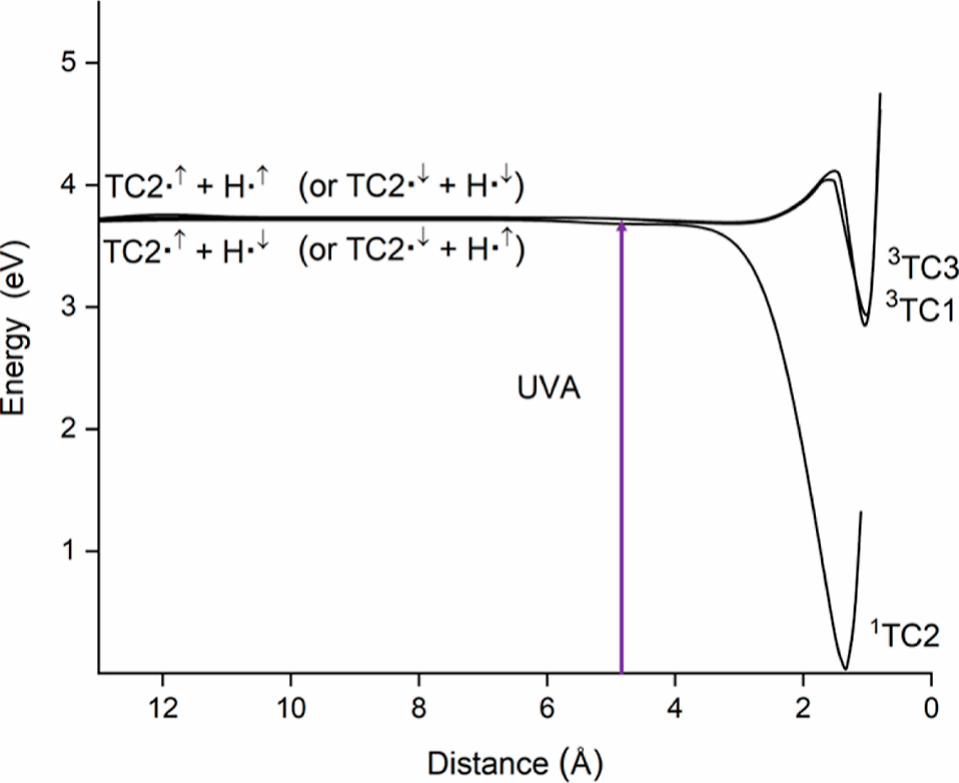
CASPT2 plots for the recombination of the radical fragments TC2^•^ + ^•^H. Triplet structures arise from the attack of the H atom to the nitrogen atoms next to the C−S bond of the thiobase. The singlet channel evolves to the amino-thiol (TC2) structure. For each plot, the TC2^•^ + ^•^H distance is relative to the atom of TC2^•^ that is attacked.

**Figure 4:**
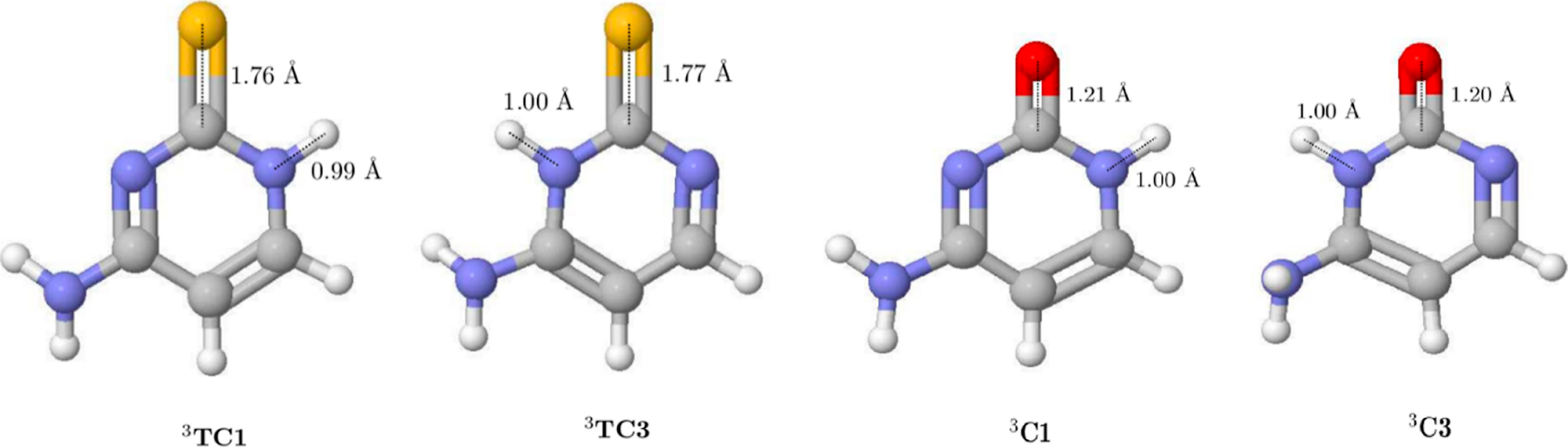
Optimized structures for the lowest-lying triplet electronic states of 2-thiocytosine and cytosine.

In Figure 3 are shown the potential energy curves for the recombination of the radicals through the triplet channels that lead to the amino-thion-N_1_H (TC1) and the amino-thion-N_3_H (TC3) tautomeric structures (shown in Figure 1). The channel leading to the attack of the H atom to the sulfur atom to yield the amino thiol (not appearing in Figure 3) does evolve to a shallow potential well, which lies only 0.05 eV below the radical asymptote. Each of the triplet pathways shown in Figure 3 reaches an energy minimum after surmounting a relatively small energy barrier. The deepest potential well was located for the structure corresponding to the amino-thion-N_1_H (TC1). This energy minimum is 2.83 eV above the ground state of 2-thiocytosine (0.9 eV below the radical asymptote), and it is reached after surmounting an energy barrier of 0.4 eV. The potential well for the amino-thion-N_3_H (TC3) structure lies 2.91 eV above the ground-state reference (0.82 eV below the radical species). The barrier that must be overcome to attain this energy minimum is 0.31 eV. The small differences in the values calculated for the transition states appearing in the plots shown in Figure 3 can be attributed to the different positions of the group NH_2_ in the tautomeric structures. The optimized structures for the triplet electronic states are shown in Figure 4. Whereas the planarity of the aromatic ring in ^3^TC1 is slightly lost (regarding the singlet counterpart), the structure for ^3^TC3 remains plane. The geometrical relaxation in ^3^TC1 could explain the slightly higher stability of this structure.

Interestingly, the energy values for these potential wells are very close to those reported by Mai et al. for the pathways leading to the formation of triplet states by invoking intersystem crossings between singlet and triplet excited electronic states of 2-thiocytosine (2.85 and 3.02 eV).^20^ However, the potential wells shown in Figure 3 correspond to different tautomeric structures and not to the same tautomer, as it is proposed in ref (20). As mentioned before, the triplet structures arise from the attack of the H atom on each of the nitrogen atoms next to the C–S bond of the molecule. According to the dominant contributions for the calculated CASSCF wave functions at the potential wells, the character of both triplet states can be assigned as ^3^*n*_s_π*. Thus, they can be responsible for the energy maximum appearing in the visible range of the transient absorption spectra reported in ref (20).

As is also seen in Figure 3, recombination of the radicals to yield the singlet lowest-lying amino-thiol tautomer TC2 takes place along a barrierless pathway. Hence, the nonradiative fast decay observed for the singlet electronic states of 2-thiocytosine can be rationalized in terms of this channel. This, in turn, accounts for the near-unity triplet yield observed for this molecule upon UVA radiation.^20^ It is important to stress that even when the reaction along the singlet channel proceeds along a barrierless pathway, this reaction is not favored over those leading to the formation of long-lived triplet states (which exhibit slight energy barriers). As discussed before, it is equally probable that the singlet radical species would be yielded as TC2^•^↑ + H^•^↓ or TC2^•^↓ + H^•^↑ upon UVA irradiation. From this, it is inferred that the proportion of radical species TC2^•^↑ + H^•^↓ is equal to the proportion of species with opposite spin TC^•^↓ + H^•^↑. Hence, regardless of the shape of the potential energy curves, the probability that fragments of the same spin approach each other to yield some of the triplet structures is always the same as that corresponding to the rebounding of species with different spin to form the singlet ground state. Thus, reactions along both spin-multiplicity channels take place during all of the photochemical process with equal probability. As the singlet ground state emerging from the radiationless decaying pathway can interact again with the UVA radiation, the reaction would reach completion after an undetermined number of excitation-decaying cycles. This might be consistent with the data provided in ref (20) for this process, as even when reaction proceeds in the femtosecond scale, the singlet–triplet conversion after 1 ps is only 74%.^20^

The global picture emerging from the proposed two-step radical reaction scheme suggests that the long-lived triplet states observed for 2-thiocytosine upon UV radiation can emerge from a photochemical process rather than a photophysical one arising from ISCs between the potential energy surfaces belonging to singlet and triplet electronic states. Instead of joining the reactants and products (of different spin-multiplicities) through a single pathway arising from intersystem-crossings between electronic states of different spin, in the proposed scheme, we consider two independent reactions: the formation and recombination of radical species. This allows the singlet and triplet channels that emerge from the radical asymptotes to evolve independently from each other to their corresponding products (as it should be expected for reactions involving only pure light-atom molecules). Although essentially different from the models widely used to explain for the shift in the spin multiplicity in this kind of systems (by invoking ISCs between electronic states of different spin multiplicity), the reaction scheme proposed in this contribution allows for rationalizing the main features of the UV transient absorption spectra recorded for 2-thiocystosine.^20^ It is worth mentioning that the picture obtained from this scheme recovers a chemical-based interpretation (the formation and recombination of radical species) for the processes leading to the ultrafast decay of the singlet excited states to the ground state of the thiobase and the formation of long-lived triplet states.

#### 2.1.1 Kinetic Aspects of the Two-Step Radical Reaction Scheme

As discussed above, the picture obtained for the process leading to the formation of long-lived triplet states and the ultrafast decay of the singlet sates using the proposed radical-reaction scheme is in good agreement with the data determined from the recorded transient absorption spectra.^20^ However, some mechanistic aspects must be addressed in order to complete the analysis of the viability of this scheme to explain this photochemical process. Mainly, the kinetics followed the recombination reactions through the triplet states. As seen in Figure 3, the triplet channels leading to the energy minima ^3^TC1 and ^3^TC3 must surmount slight energy barriers. We used the canonical transition-state expression (eq 2) to estimate the rate constant for the reaction that evolves to the most stable structure ^3^TC1.
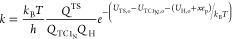
2In this equation, *Q*^TS^ represents the partition function for the transition state and *Q*_TC1N_ and *Q*_H_ represent the corresponding partition functions for the reactants. A similar notation was used for the free energy *U*_x,o_ (values for these energies include the ZPE correction). *k*_B_ is the Boltzmann constant, *h* the Planck constant, and *T* the absolute temperature. To analyze the effect of UVA electromagnetic radiation on the kinetics of the reaction leading to the rebounding of the radicals, the energy of the incident photons (3.49 eV at the maximum of the transient absorption spectra) was distributed (in different percentages) between the fragments TC1_N_^•^ and H^•^. The term *xe*_p_ in eq 2 is the fraction of the photon energy gained by the H^•^ radical. It is assumed that the fraction of the energy absorbed by the radical fragment TC1_N_^•^ is distributed through all of the radical structure and that this energy remains when the transition state structure is formed, thus having no effect on the values of the rate constant. According to the data provided in Table 3, the values for the kinetic constant vary significantly when *xe*_p_ increases.

**Table 3:** Rate Constants Calculated for the Conversion of Different Proportions of the UVA Incident Energy into Hydrogen Internal Energy^a^

energy fraction of the photon	*k* (M^–1^ s^–1^)	*k* (s^–1^)
0.00	1.11 × 10^–3^	5.55 × 10^–6^
0.10	8.83 × 10^2^	4.41
0.20	6.98 × 10^5^	3.49 × 10^3^
0.30	5.51 × 10^14^	2.75 × 10^12^
0.35	4.90 × 10^17^	2.45 × 10^15^
0.40	4.35 × 10^20^	2.17 × 10^18^

a Value for the energy of the incident photon was taken at the maximum of the transient absorption spectra reported in ref (20) (3.49 eV). For calculation of the rate constants in the third column, the concentration of 2-thiocytosine in aqueous solution was taken as 0.005 M (the lowest concentration used in ref (20) to determine the transient spectra).

Whereas in the absence of UVA radiation, the rate constant has the value 5.55 × 10^–6^ s^–1^, an increment of 0.35*e*_p_ raises the constant value to 2.45 × 10^15^ s^–1^. Thus, although the distribution percentages of the incident energy between the two radical species are unknown, a relatively small energy transfer toward hydrogen might lead to a significant increase in the reaction rate. This is consistent with the ultrafast triplet-state population observed for this process. In fact, values in Table 3 suggest that the reaction might proceed without an energy barrier at higher-energy percentages (however, it is important keep in mind that a fraction of the energy of the photons will be transferred to the radical species TC1_N•_).

For comparison, we used the expression obtained from the semiclassical Marcus theory (eq 1) to evaluate the intersystem-crossing rate at the lowest-lying crossing between the singlet and triplet potential energy curves arising from the elongation of the C–S distance of 2-thiocytosine (as discussed by Mai et al., the excited electron seems to be localized on the thio group upon UV radiation; thus, the C–S distance might correspond to the distortion coordinate). A plot highlighting the ISC between these potential curves and details on the calculation of the ISC rate are provided in the Supporting Information. The calculated ISC rate is 3.33 × 10^12^ s^–1^. This value seems to be small for a process taking place in the femto-second scale. However, this is not the main disadvantage of the description made in ref (20) for the interaction of the thiobase with UVA radiation, as the value calculated for the hopping probability from the singlet to the triplet state using the Landau–Zener model is only 0.1072 (details on the use of this model are provided in the Supporting Information). The small value calculated for this probability suggests that it is highly unlikely that a near-unity singlet–triplet conversion could take place through the intersystem crossing between the potential energy curves belonging to these electronic states, as it is proposed in ref (20).

### 2.2 Cytosine

Photoexcitation of the natural nucleobases with UVA radiation does not produce long-lived triplet structures. In all the cases, a fast nonradiative decay to the singlet ground state is the only process observed.^5−10^ However, excited triplet electronic states have been detected for some of them at higher energies.^11,12^ Particularly, Abouaf et al. have investigated the excitation of the lowest electronic states of cytosine by electron energy loss spectroscopy.^11^ The EEL spectra recorded by these authors show the existence of shoulders at energy losses of 3.50 and 4.25 eV. These shoulders have been attributed to the lowest-lying triplet states of the cytosine. The analysis of the optical absorption spectrum of cytosine in a solution of neutral water reinforces these assignments.^12^

**Figure 5:**
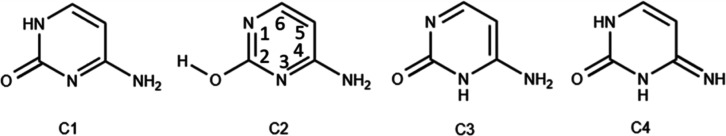
Structures for the lowest-lying singlet tautomers of cytosine: the amino-oxo-N1H (C1), amino-hydroxo (C2), amino-oxo-N3H (C3), and imino-oxo (C4) forms. As for 2-thiocytosine, the pyrimidine atom-numbering scheme has been used in the most stable tautomer C2.

A reaction scheme such as that used to describe the photochemical pattern of 2-thiocytosine can be applied to rationalize these experimental data. In Table 3 are provided the energy values for the singlet and triplet radical species arising by hydrogen abstraction from the NH_2_ and OH groups of the cytosine; the energies for the lowest-lying tautomers of cytosine are also collected in this table The structures for these tautomers are shown in Figure 5.

**Figure 6:**
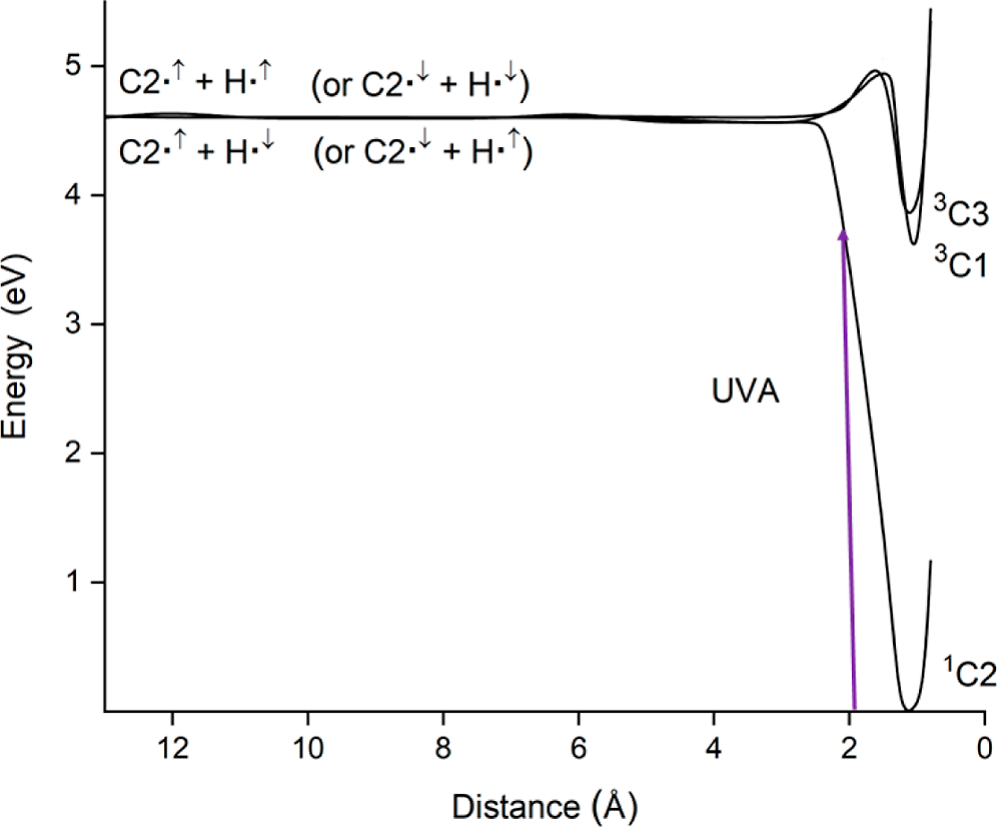
CASPT2 plots for the recombination of the radical fragments C2^•^ + ^•^H. Triplet structures arise from the attack of the H atom to the nitrogen atoms next to the C−O bond of the nucleobase. The singlet channel evolves to the amino-hydroxo (C2) structure. For each plot, the C2^•^ + ^•^H distance is relative to the atom of C2^•^ that is attacked.

Interestingly, the energy values for the radical fragments yielded by hydrogen abstraction from the NH_2_ and OH groups of the cytosine are very close to the energy of the highest shoulder recorded in the EEL spectra (4.25 eV).^11^ Thus, this shoulder might correspond to the energy involved in the homolytic breaking of the O–H bonds of the nucleobase. Once the radicals are yielded, they can recombine in a second reaction. In Figure 6, plots for the electronic states emerging from the recombination of the lowest-lying radical species are shown.

As discussed in the previous section for 2-thiocytosine, recombination of the radical fragments can take place along both the singlet and triplet channels. Rebounding through the singlet pathway leads to the ground state of the cytosine through a barrierless pathway. Thus, this channel can explain for the ultrafast nonradiative decay observed when this molecule is exposed to UV radiation.^31^ As is also seen in Figure 6, the recombination of the radical species along the triplet channels yields the amino-oxo-N_1_H (C1) and the amino-oxo-N_3_H (C3) tautomers. The potential energy wells for these structures lie 3.29 and 3.5 eV above the ground state reference, respectively (optimized structures for these electronic states are shown in Figure 4). These energies are in good agreement with the experimental values reported in refs (11 and 12) for the energy of the lowest lying triplet state (3.5 eV).

Plots appearing in Figures 3 and 6 exhibit similar trends (reflecting the fact that the nucleobase and the thiobase differ from each other in only one equivalent atom that belongs to elements of the same group). However, the photochemical patterns observed for these molecules when they are irradiated with UVA light differ from each other: whereas for 2-thiocytosine are observed both a nonradiative fast decay to the singlet ground state and the formation of long-lived triplet states, for cytosine only a fast decay to the ground state is detected. This different behavior can be explained in terms of the energy values provided in Tables 1 and 4 for the radical fragments yielded from these molecules by hydrogen abstraction. The energy value appearing in Table 1 for the radical fragments produced by homolytic breaking of the S–H bond of thiocytosine (3.43 eV) lies below the UVA upper limit (around 3.89 eV). As discussed in the preceding section, this allows an explanation for the long-lived triplet states observed for this thiobase. On the other hand, the energy values provided in Table 4 for the radical species yielded by hydrogen abstraction of the NH_2_ and OH groups of cytosine are all above that limit. Thus, the energy supplied by this kind of radiation is not enough to break the O–H bonds of the cytosine molecule. As schematically shown in Figure 6 (violet line), when the nucleobase is exposed to UVA radiation, only the singlet pathway falling to the ground state is reached; this explains the absence of long-lived triplet states as well as the fast decay to the ground state observed when cytosine is irradiated with UVA light. (However, it is important to note that triplet structures can be obtained when this nucleobase is exposed to UVB and UVC radiations, as in this case, the radiation energy is high enough to induce the homolytic breaking of the O–H bonds of the molecule.)

**Table 4:** Energies for the Lowest-Lying Singlet Tautomers of Cytosine [the Amino-oxo-N_1_H (C1), Amino-hydroxo (C2), Amino-oxo-N_3_H (C3), and Imino-oxo (C4) Forms] and Energies for the Singlet and Triplet Radical Fragments Obtained by Hydrogen Abstraction of the NH_2_ and OH Groups of the Cytosine

	CASPT2 energy (au)	CASSCF-ZPE (au)	CASPT2 + ZPE (au)	relative energy (eV)	relative energy (kcal/mol)
^(T)^C1_NH__^•^_+ H^•^	–393.6885329	0.089094	–393.5994389	4.26	98.3
^(S)^C1_NH__^•^_+ H^•^	–393.6886903	0.089094	–393.5995963	4.26	98.2
^(T)^C2^•^ + H^•^	–393.6910554	0.089393	–393.6016624	4.20	96.9
^(S)^C2^•^ + H^•^	–393.6916239	0.089393	–393.6022309	4.19	96.5
C3	–393.8455581	0.104452	–393.7411061	0.41	9.4
C4	–393.8559784	0.104656	–393.7513224	0.13	2.9
C1	–393.8563302	0.104618	–393.7517122	0.12	2.7
C2	–393.8605199	0.104493	–393.7560269	0	0

## 3 Conclusions

A computational CASSCF-CASPT2 study was carried out to analyze the possible role of radical species in the formation of the long-lived triplet states observed for 2-thiocytosine upon UVA irradiation. It is predicted that the radical species obtained by expelling the hydrogen atom of the SH group of the molecule can be yielded at the UVA and UVB radiations employed to record the transient absorption spectra reported in ref (20). Once the radical species are formed, their recombination through the triplet channels arising from the attack of the hydrogen atom to the nitrogen atoms next to the C–S bond of the molecule evolves to the amino-thion-N_1_H (TC1) and the amino-thion-N_3_H (TC3) tautomeric structures. The rebounding through the singlet channel yields the lowest-lying tautomer of 2-thiocytosine (the amino-thiol tautomer) through a barrierless pathway. These results shed light on the formation of long-lived triplet states and the ultrafast decay observed for the excited singlet state when 2-thiocytosine is exposed to UVA radiation, without invoking interactions between the electronic states of different spin-multiplicities (intersystem-crossings).

The picture obtained from a similar study for cytosine shows that the homolytic breaking of the O–H bond of this molecule is not feasible under UVA radiation. This cancels the possibility that triplet states can be obtained via the formation and recombination of radical species when cytosine is exposed to this light. However, the radical fragments arising from the hydrogen abstraction of the O–H group of the nucleobase can be produced at higher radiation frequencies (in the UVB and UVC regions), switching the recombination channel that leads to the formation of triplet states, consistent with the experimental observations.

Hopefully, the results emerging from this study could inspire future experimental and theoretical investigations on the role that radical species could play in these kinds of photochemical reactions.

## 4 Methods

A gas-phase-type study was conducted to investigate the possible role of radical species in the formation of the long-lived triplet structures observed when 2-thiocytosine is exposed to UV irradiation. For this, the structures of the four low-lying singlet tautomers of 2-thiocytosine (shown in Figure 1) were optimized through CASSCF(14,10)/ANO-S-VDZP calculations.^32,33^ The energies of the optimized structures were revaluated at the MS-CASPT2/ANO-S-VDZP level of calculation (averaging two states). Likewise, energies for the singlet and triplet radical asymptotes obtained by hydrogen abstraction of the groups SH and NH_2_ of 2-thiocytosine (TC2^•^ + H^•^ and TC1_NH^•^_ + H^•^, respectively) were calculated through CASSCF geometry optimization calculations followed by single-point energy evaluation at the MS-CASPT2 level.

Potential energy curves for the recombination of the radical fragments TC2^•^ + H^•^ to yield the triplet state of the amino-thion-N_1_H and the amino-thion-N_3_H tautomeric structures as well as the lowest-lying singlet tautomer (amino-thiol) were investigated through partial geometry optimization calculations at fixed distances of the radical species. For each plot, the energy calculated at each distance was revaluated at the MS-CASPT2 level of theory. All of the stationary points located along these curves were characterized as energy minima or transition states through frequency analysis calculations. For CASPT2 calculations, an imaginary level shift of 0.3 au and a default IPEA shift of 0.25 au were used.

For comparison, a similar study was carried out for the cytosine molecule (the corresponding canonical base). Particularly, the singlet and triplet pathways that emerge from the recombination of the radical fragments arising from the homolytic breaking of the O–H bond of this molecule were investigated. As the same type of basis sets were used for both 2-thiocytosine and the cytosine molecules (ANO-S-VDZP), the number of CSF́s expanded by the active space (14,10) was also the same: 4950 for the tautomers and 6930 for the separated radical fragments. For the two lowest-lying states of both molecules (TC1 and TC2 for thiocytosine and CI and C2 for cytosine), the active space orbitals used to describe both regions of the potential energy surfaces (the tautomers and the radical fragments) appear in Figures S3–S6. Most of them exhibit dominant contributions of the *p*_*z*_ atomic functions (mainly centered on the carbon and nitrogen atoms) perpendicular to the plane of the molecule or the radical fragment (TC2^•^ or C2^•^). As seen in Figures S4 and S6, orbitals for describing the recombination of the radical species are also included in the active space.

All the calculations were carried out using the package of programs MOLCAS 8.4.^34^

## Supporting Information Available

Structure showing the atom numbering used to analyze the dominant molecular contributions, CASPT2 energies, ZPEs (CASSCF) and Cartesian coordinates for the stationary states discussed herein, details of the calculation of the ISC rate and the Landau–Zener hopping probability for the singlet–triplet states, and active space orbitals used to describe the two regions of the potential energy surfaces investigated (PDF)
