# Supplementary material for: Disruptive Model That Explains for the Long-Lived Triplet States Observed for 2-Thiocytosine upon UVA Radiation
Source: ACS Omega. 2024 Mar 8;9(11):13059–66. doi: 10.1021/acsomega.3c09471 (PMC10955585; doi:10.1021/acsomega.3c09471)

SUPPORTING INFORMATION

Disruptive Model That Explains for the Long-Lived Triplet States Observed for 2-Thiocytosine upon UVA Radiation.

Jorge Baños, Alejandro Avilés and Fernando Colmenares*

Departamento de Física y Química Teórica, Facultad de Química, Universidad Nacional Autónoma de México, CDMX 04510, Mexico.

E-mail: colmen@unam.mx

Contents

Figure S1. Plots at the crossing of the potential energy curves belonging to the lowest-lying singlet and triplet excited states of 2-thiocytosine.

Calculation of the rate constant at the intersystem crossing (ISC) between the lowest-lying excited singlet and triplet electronic states of 2-thiocytosine.

Evaluation of the Landau-Zener hopping probability between the singlet and triplet states.

Figure S2. Structure showing the atom numbering used for the CASSCF and CASPT2 calculations.

Table S1. CASPT2 energies, Zero-point energies (CASSCF) and cartesian coordinates for the stationary states appearing in figures and tables.

Figures S3-S6. Active space orbitals used to describe the low-lying tautomers and the radical species investigated for 2-thiocytosine and cytosine.

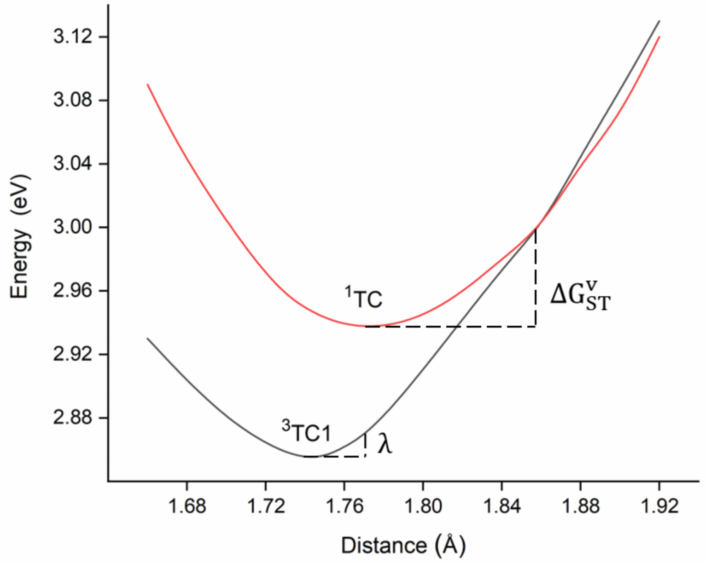

Figure S1. Plots at the crossing of the potential energy curves belonging to the lowest-lying singlet and triplet excited states of 2-thiocytosine. The values of the reorganization energy and the free energy appearing in equation 1 of the main text are schematically shown (see below).

For comparison, it is mentioned that the energy values at the potential wells in Figure S1 are practically the same that those appearing in Figure 4 of reference 20 for the singlet and triplet electronic states.

Calculation of the rate constant at the intersystem crossing (ISC) between the lowest lying excites singlet and triplet electronic states.

The rate constant at the ISC shown in Figure S1 was calculated using equation 1 in the main text:

$$k_{ISC} = \frac{2\pi}{\hbar}|V_{SOC}|^2 \frac{1}{\sqrt{4\pi\lambda k_B T}} exp\left\{-\frac{(\Delta G^{v}_{ST})^2}{4\lambda k_B T}\right\}$$

As used in reference 21, the reorganization energy λ was approximated as $E_T(R_{min\ S}) - E_T(R_{min\ T})$ and ΔG^{v}_{ST} as $E_{T(CP)}(R_{min\ S})$ - $E(R_{min\ S})$. Thus, λ = 3.051x10^{-21} J and ΔG^{v}_{ST}= 1.094x10^{-20} J. The calculated value for the spin-orbit constant at the crossing point is V_{soc} = 2.76x10^{-21} J.

Evaluation of the Landau-Zener hopping probability

The hopping probability was calculated using the equations:

$$P^{ISC} = 1 - P^{LZ}$$

$$P^{LZ} = e^{\left[-\frac{2\pi V_{SOC}^2}{\hbar v|F_T - F_S|}\right]}$$

where v is the velocity and F_T and F_S are the slopes of the potentials at the crossing point. Equivalently,

$$P^{LZ}(U - E_{CP}) = e^{\left[\frac{2\pi V_{SOC}^2}{\hbar|F_T - F_S|}\sqrt{\frac{\mu_x}{2(U - E_{CP})}}\right]}$$

Where $\mu_x = 1.45x10^{-26}kg$ is the reduced mass of the distortion coordinate (see main text), V_{SOC} = 2.76x10^{-21} J, U is the total internal energy and E_{CP} is the energy at the crossing point (U – E_{CP}) = 4.244x10^{-19} J. The values for the slopes F_T = 0.072 and F_S = 0.012 were calculated at the crossing point shown in Figure S1.

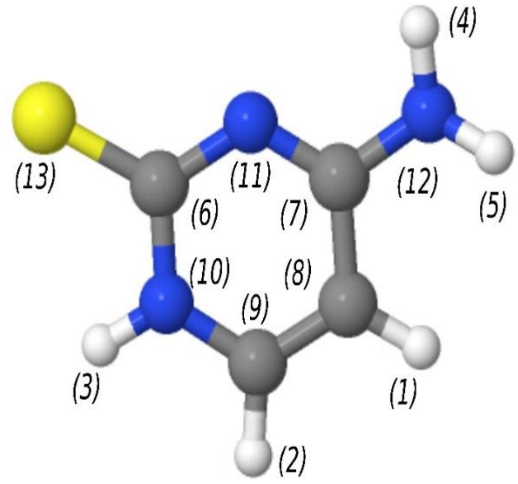

Figure S2. Atom numbering used for the CASSCF and CASPT2 calculations. This numbering must be used as reference for the coordinates provided in Table S1 for 2-thiocytosine and cytosine.

Table S1. CASPT2 energies, Zero-point energies (CASSCF) and cartesian coordinates for the stationary states appearing in figures and tables.

(Table 1, Figure 1):

Tautomer TC1 (singlet)
Energy (CASPT2) = -716.44954198
ZPE(CASSCF(14,10)) = 0.102291

H1	1.93223462	3.56657289	0.26008290
H2	-0.59131207	3.43724426	0.23067573
H3	-1.56585071	1.30730280	0.02848718
H4	3.88027055	0.49556663	-0.19259419
H5	3.93135219	2.18364489	-0.19882125
C6	0.10789258	0.17831623	-0.07304208
C7	2.08983651	1.36643034	0.04102593
C8	1.40184769	2.63496786	0.15914638
C9	0.05078964	2.57414774	0.14928309
N10	-0.56717409	1.36627048	0.03544128
N11	1.47790642	0.22796858	-0.07000549
N12	3.45680986	1.35595743	0.09851797
S13	-0.74057862	-1.24203706	-0.20836942

(Table 1, Figures 1 and 2):

Tautomer TC2 (singlet)
Energy (CASPT2) = -716.45381740
ZPE(CASSCF(14,10)) = 0.098170

H1	1.48341827	3.38568043	-0.20797314
H2	-1.01446003	3.58973281	-0.29178204
H3	2.98401948	0.04241057	-0.00461093
H4	3.30868867	1.65122420	-0.32986173
H5	-2.91256179	-0.43374689	0.14091554
C6	-0.74682657	0.40469230	0.09000998
C7	1.36573137	1.22101587	0.03248380
C8	0.84304980	2.52117993	-0.12955105
C9	-0.53273565	2.62816238	-0.17007344
N10	-1.34849553	1.56699468	-0.05867323
N11	0.56754760	0.17462249	0.14004833
N12	2.72224045	0.99767108	0.14987288
S13	-1.74069667	-1.06157331	0.24272310

(Table 1, Figure 1):
Tautomer TC3 (singlet)
Energy (CASPT2) = -716.43803057
ZPE(CASSCF(14,10)) = 0.102006

H1	1.53537864	3.03523958	0.92579694
H2	-0.32228625	2.77358855	-0.76217202
H3	4.16044371	0.58302827	1.02823859
H4	3.51816473	1.84958517	1.86568981
H5	2.66054226	-0.82594695	-0.16047059
C6	1.08620133	-0.14107101	-1.22529137
C7	2.28228118	1.07813415	0.49375372
C8	1.43106251	2.13069370	0.35101528
C9	0.38789453	1.96638409	-0.61765579
N10	0.21594851	0.92000389	-1.36445326
N11	2.07903548	-0.01989072	-0.28420898
N12	3.32042847	0.97976183	1.40953713
S13	0.96942933	-1.52324573	-2.12860859

Tautomer TC4 (singlet)
Energy (CASPT2) = -716.44775059
ZPE(CASSCF(14,10)) = 0.102496

H1	1.77280660	3.27451959	0.17593799
H2	-0.52744319	2.31240124	0.31552541
H3	-0.80594918	-0.01782395	0.05802353
H4	4.09059005	2.59986471	-0.17147096
H5	3.09805598	-0.68468876	-0.46285433
C6	1.10997162	-0.55177235	-0.22296576
C7	2.72002029	1.31277167	-0.17381931
C8	1.59200294	2.21837380	0.06555542
C9	0.34913669	1.70871317	0.14211708
N10	0.11992525	0.35132116	-0.00087110
N11	2.35590774	-0.03239493	-0.29719467
N12	3.95458457	1.60599507	-0.27803305
S13	0.79913172	-2.17597154	-0.38984556

(Table 1, Figure 3):

Radical fragments $^{(T)}$TC1$_{NH}$· + H·
Energy (CASPT2) = -716.28785221
ZPE (CASSCF(14,10)) = 0.087516

H1	1.76079386	3.76811564	0.39845246
H2	-0.73005733	3.42955982	0.40340544
H3	-1.54380951	1.21516878	0.06300050
H4	3.86735199	0.84426471	-0.17902210
H5	12.87154050	10.64810803	1.09024002
C6	0.27815695	0.30146595	-0.16468607
C7	2.18181515	1.64023452	0.01098668
C8	1 .31268708	2.80086472	0.23960018
C9	-0.02258176	2.63126578	0.24427589
N10	-0.56039015	1.37145426	0.03005318
N11	1.56311879	0.38257836	-0.18537655
N12	3.45480816	1.75340984	-0.00952779
S13	-0.48350607	-1.26334906	-0.39997319

Radical fragments $^{(S)}$TC1$_{NH}$· + H·
Energy (CASPT2) = -716.28857467
ZPE (CASSCF(14,10)) = 0.087516

H1	1.76079386	3.76811564	0.39845246
H2	-0.73005733	3.42955982	0.40340544
H3	-1.54380951	1.21516878	0.06300050
H4	3.86735199	0.84426471	-0.17902210
H5	12.87154050	10.64810803	1.09024002
C6	0.27815695	0.30146595	-0.16468607

C7	2.18181515	1.64023452	0.01098668
C8	1.31268708	2.80086472	0.23960018
C9	-0.02258176	2.63126578	0.24427589
N10	-0.56039015	1.37145426	0.03005318
N11	1.56311879	0.38257836	-0.18537655
N12	3.45480816	1.75340984	-0.00952779
S13	-0.48350607	-1.26334906	-0.39997319

Radical fragments $^{(T)}$TC2· + H·
Energy (CASPT2) = -716.31694681
ZPE(CASSCF(14,10)) = 0.088322

H1	1.93577843	3.54241533	0.26065522
H2	-0.56919083	3.42357286	0.21671374
H3	3.87268974	0.46451402	-0.22551856
H4	3.97102490	2.13566290	-0.20137269
H5	-13.57403100	0.21946200	-0.16586200
C6	0.13068270	0.29640575	-0.05941050
C7	2.11241665	1.37911699	0.05445018
C8	1.41699852	2.60192337	0.16064203
C9	0.03760888	2.53043055	0.14066881
N10	-0.62643160	1.36869415	0.03144922
N11	1.46207528	0.23331616	-0.05620137
N12	3.48807559	1.32185514	0.12341892
S13	-0.70873484	-1.25980740	-0.21069709

Radical fragments $^{(S)}$TC2· + H·
Energy (CASPT2) = -716.31769252
ZPE (CASSCF(14,10)) = 0.088322

H1	1.93577843	3.54241533	0.26065522
H2	-0.56919083	3.42357286	0.21671374
H3	3.87268974	0.46451402	-0.22551856
H4	3.97102490	2.13566290	-0.20137269
H5	-13.57403100	0.21946200	-0.16586200
C6	0.13068270	0.29640575	-0.05941050
C7	2.11241665	1.37911699	0.05445018
C8	1.41699852	2.60192337	0.16064203
C9	0.03760888	2.53043055	0.14066881
N10	-0.62643160	1.36869415	0.03144922
N11	1.46207528	0.23331616	-0.05620137
N12	3.48807559	1.32185514	0.12341892
S13	-0.70873484	-1.25980740	-0.21069709

(Figure 3):

Energy minimum ^{T}TC1
Energy (CASPT2) = -716.34993823
ZPE(CASSCF(14,10)) = 0.097261

H1	1.94952039	3.55673366	0.23397676
H2	-0.59978664	3.46752752	0.30836339
H3	-1.57858666	1.27669872	-0.05836988
H4	3.83387068	0.47773976	-0.31977539
H5	4.00397256	2.11143699	-0.17426420
C6	0.16137671	0.21707876	-0.04414646
C7	2.11648489	1.42990399	0.04874355
C8	1.42443272	2.61672876	0.14869482
C9	0.02099728	2.61069457	0.11983276
N10	-0.59935837	1.35186421	0.11394159
N11	1.44568863	0.19729471	-0.05649448
N12	3.50795377	1.30273375	0.15094668
S13	-0.73054136	-1.29408232	-0.21162115

Transition state ^{3}TC1
Energy (CASPT2) = -716.30223541
ZPE(CASSCF(14,10)) = 0.091151

H1	-0.13013712	2.37788083	0.67991548
H2	-1.41567222	0.30112692	0.14049823
H3	3.48436309	2.47248029	0.07019957
H4	2.09758238	3.41204791	0.24453584
H5	-0.26036890	-1.18887360	-1.58250039
C6	1.61401231	-0.79465752	-0.24041844
C7	1.79065708	1.41133701	0.19998657
C8	0.36143753	1.47024758	0.36644279
C9	-0.33597830	0.33503134	0.10165212
N10	0.28469510	-0.84100477	-0.26451916
N11	2.38348980	0.30392108	-0.10194878
N12	2.54493226	2.53407741	0.41346835
S13	2.45362833	-2.30087465	-0.60630646

Energy minimum ^{3}TC3
Energy (CASPT2) = -716.34707480
ZPE(CASSCF(14,10)) = 0.098649

H1	1.55865045	2.99813277	0.93327443

H2	-0.35453177	2.77456526	-0.74085209
H3	4.26605668	0.83258058	0.91960099
H4	3.43215859	1.75841350	1.98743640
H5	2.38446699	-0.92124588	0.08708091
C6	1.10846909	-0.06486151	-1.24572723
C7	2.32990657	1.07845146	0.45697908
C8	1.44345484	2.10755627	0.33342574
C9	0.37439956	1.99539332	-0.59548125
N10	0.25853048	0.87635492	-1.40005063
N11	2.20427246	-0.04872763	-0.37699649
N12	3.37890429	0.96247300	1.37940892
S13	0.93978614	-1.54282132	-2.20692788

Transition state ^{3}TC3
Energy (CASPT2) = -716.30540810
ZPE(CASSCF(14,10)) = 0.091221

H1	1.50682377	2.99531417	0.94976431
H2	-0.45027313	2.68988462	-0.59780102
H3	4.19030583	0.53113039	0.94263901
H4	3.67928813	1.97195805	1.63973367
H5	2.12664273	-1.16666447	0.53445232
C6	1.20257839	0.00948280	-1.26923400
C7	2.35322928	1.11431491	0.37998019
C8	1.41449716	2.11214520	0.33639987
C9	0.33314250	1.94603921	-0.53850640
N10	0.22647180	0.90016768	-1.34969111
N11	2.22951285	0.00618467	-0.40609661
N12	3.41725546	1.09290540	1.24202834
S13	1.09504959	-1.39659790	-2.33249768

(Table 4, Figure 2S):

Tautomer C1 (singlet)
Energy (CASPT2) = -393.8563302
ZPE(CASSCF(14,10)) = 0.104618

H1	1.93102560	3.51547819	0.27239691
H2	-0.58660470	3.41596514	0.22646819
H3	-1.58986619	1.28479438	0.01489690
H4	3.87827537	0.45875676	-0.18889157
H5	3.90832761	2.13907711	-0.22638955
C6	0.08658670	0.13050587	-0.07892216

C7	2.07923426	1.31004961	0.04292529
C8	1.39119895	2.59045911	0.16278002
C9	0.04333975	2.54370470	0.14480966
N10	-0.59206551	1.33901769	0.02368563
N11	1.47719732	0.17743356	-0.07414978
N12	3.45395340	1.31536218	0.11277728
O13	-0.52557799	-0.89625119	-0.17365884

(Table 4, Figures 5 and 2S):

Tautomer C2 (singlet)
Energy (CASPT2) = -393.8605199
ZPE(CASSCF(14,10)) = 0.104493

H1	0.36415113	4.49590045	-1.18115406
H2	-2.10592047	4.52361657	-0.74241718
H3	2.10943713	1.26956098	-1.26491005
H4	2.24264301	2.89103519	-1.66130924
H5	-3.08483725	0.43969835	0.01875419
C6	-1.52635951	1.37830653	-0.40036713
C7	0.45240962	2.33262593	-0.90238967
C8	-0.18282291	3.59233138	-0.96398971
C9	-1.53917812	3.60204450	-0.71677309
N10	-2.23172222	2.48765514	-0.42886348
N11	-0.22227530	1.23385041	-0.62194550
N12	1.81553015	2.20754105	-1.06864889
O13	-2.16851223	0.24156031	-0.12215669

(Table 4, Figure 2S):

Tautomer C3 (singlet)
Energy (CASPT2) = -393.8455581
ZPE(CASSCF(14,10)) = 0.104452

H1	1.53803651	3.03548404	0.94098984
H2	-0.31856356	2.79747191	-0.74842036
H3	4.17008805	0.60874841	1.02278466
H4	3.50879584	1.84462190	1.88821792
H5	2.65031248	-0.83169422	-0.15011900
C6	1.07360427	-0.13427349	-1.23026841
C7	2.29094820	1.08980299	0.49179352
C8	1.44006231	2.13633364	0.35671301
C9	0.38439575	1.98061981	-0.61733309
N10	0.19842398	0.94862110	-1.36777319

N11	2.10093871	-0.00911588	-0.30069933
N12	3.32345208	0.98088718	1.41511237
O13	0.96402984	-1.14124258	-1.86982709

Tautomer C4 (singlet)
Energy (CASPT2) = -393.8559784
ZPE(CASSCF(14,10)) = 0.104656

H1	2.01571979	3.52675145	0.34579495
H2	-0.43419463	3.09452850	0.33475136
H3	-1.21090789	0.88796962	-0.04218559
H4	2.50347624	-0.60449928	-0.40154759
H5	4.13968071	2.39975132	0.08391455
C6	0.56583088	-0.05785579	-0.25296357
C7	2.54233565	1.41849184	-0.04238775
C8	1.61869679	2.53903887	0.18151953
C9	0.29245870	2.31323170	0.17680842
N10	-0.22568483	1.04733165	-0.03303670
N11	1.91484959	0.18923081	-0.23968053
N12	3.81540180	1.45753752	-0.07260667
O13	0.10611719	-1.14969909	-0.43954193

(Table 4):
Radical fragments $^{(T)}C1_{NH}\cdot$ + H·
Energy (CASPT2) = -393.6885329
ZPE (CASSCF(14,10)) = 0.089094

H1	1.74320151	3.73305857	0.39723841
H2	-0.76594726	3.37817225	0.39891694
H3	-1.55510581	1.17817356	0.03540024
H4	3.86475920	0.82623624	-0.17494810
H5	12.87154050	10.64810803	1.09024002
C6	0.21233736	0.21145741	-0.18147253
C7	2.11388419	1.59049233	0.00811576
C8	1.28578970	2.77158772	0.23917801
C9	-0.04817365	2.58910481	0.24091312
N10	-0.56683990	1.32841206	0.03008014
N11	1.60827794	0.38937517	-0.18958966
N12	3.46021484	1.74060178	-0.00234432
O13	-0.27334879	-0.87080867	-0.35333818

Radical fragments $^{(S)}C1_{NH}\cdot$ + H·
Energy (CASPT2) = -393.6886903
ZPE (CASSCF(14,10)) = 0.089094

H1	1.74320151	3.73305857	0.39723841
H2	-0.76594726	3.37817225	0.39891694
H3	-1.55510581	1.17817356	0.03540024
H4	3.86475920	0.82623624	-0.17494810
H5	12.8715405	10.64810803	1.09024002
C6	0.21233736	0.21145741	-0.18147253
C7	2.11388419	1.59049233	0.00811576
C8	1.28578970	2.77158772	0.23917801
C9	-0.04817365	2.58910481	0.24091312
N10	-0.56683990	1.32841206	0.03008014
N11	1.60827794	0.38937517	-0.18958966
N12	3.46021484	1.74060178	-0.00234432
O13	-0.27334879	-0.87080867	-0.35333818

(Table 4, Figure 5):

Radical fragments $^{(T)}$C2· + H·
Energy (CASPT2) = -393.6910554
ZPE(CASSCF(14,10)) =0.089393

H1	1.91958229	3.49615727	0.27667999
H2	-0.60100267	3.40248970	0.22322491
H3	3.90274587	0.45292509	-0.17982198
H4	3.93767314	2.12449053	-0.25503846
H5	-13.2158390	4.76836701	0.24601402
C6	0.09133728	0.19204930	-0.07431636
C7	2.10805502	1.30874297	0.04492404
C8	1.39747056	2.55950570	0.16493746
C9	-0.01460422	2.49456299	0.14186671
N10	-0.66975274	1.37003640	0.02908831
N11	1.48802075	0.18305206	-0.07177167
N12	3.48625291	1.31430509	0.12138317
O13	-0.50181660	-0.85803831	-0.17649219

Radical fragments $^{(S)}$C2· + H·
Energy (CASPT2) = -393.6916239
ZPE (CASSCF(14,10)) = 0.089393

H1	1.91958229	3.49615727	0.27667999
H2	-0.60100267	3.40248970	0.22322491
H3	3.90274587	0.45292509	-0.17982198
H4	3.93767314	2.12449053	-0.25503846
H5	-13.2158390	4.76836701	0.24601402

C6	0.09133728	0.19204930	-0.07431636
C7	2.10805502	1.30874297	0.04492404
C8	1.39747056	2.55950570	0.16493746
C9	-0.01460422	2.49456299	0.14186671
N10	-0.66975274	1.37003640	0.02908831
N11	1.48802075	0.18305206	-0.07177167
N12	3.48625291	1.31430509	0.12138317
O13	-0.50181660	-0.85803831	-0.17649219

(Figure 5):

Energy minimum ^{T}C1
Energy (CASPT2) = -393.7313887
ZPE(CASSCF(14,10)) = 0.096267

H1	2.38573900	4.11790301	1.12611438
H2	-0.12119849	3.40885087	1.56081775
H3	-0.79483778	1.35800903	0.45328784
H4	4.57999285	2.07005970	-0.98785797
H5	4.50448404	3.49743200	-0.02951831
C6	0.95064746	0.90948535	-0.45878456
C7	2.74678683	2.39457114	-0.12610102
C8	1.97616860	3.22306323	0.68748192
C9	0.56010589	2.85459352	0.94778724
N10	0.14073628	1.68875278	0.33713179
N11	2.27929594	1.27984234	-0.68814727
N12	4.06371870	2.68807609	-0.40138023
O13	0.51895055	-0.09666777	-0.96244163

Transition state ^{3}C1
Energy (CASPT2) = -393.6746041
ZPE(CASSCF(14,10)) = 0.092015

H1	0.10874659	-0.31263027	-0.54074503
H2	-2.03019746	1.08854438	-0.96052061
H3	2.76320335	1.11111772	1.55540949
H4	1.98310697	-0.32888432	1.12555666
H5	-1.22652912	3.62590635	-1.74251378
C6	0.09069669	3.42654511	0.14342677
C7	1.08971588	1.38116017	0.49264765
C8	0.01770355	0.70910215	-0.20906703
C9	-1.14306443	1.52932773	-0.52817413
N10	-1.05533509	2.83523518	-0.48679206
N11	1.08718617	2.64418037	0.70002679

N12	2.14760698	0.63587465	0.92304749
O13	0.13527354	4.62316619	0.19294303

Energy minimum ^{3}C3
Energy (CASPT2) = -393.728053
ZPE(CASSCF(14,10)) = 0.100848

H1	1.91452250	3.19923388	0.47718789
H2	-0.27623082	2.84724588	-0.62436388
H3	3.95792939	1.79220249	1.51787261
H4	2.95228154	0.61634429	2.07181249
H5	2.73147559	-0.87315051	-0.36796235
C6	0.95848549	-0.19894237	-1.06575789
C7	2.65381948	1.12435357	0.13233542
C8	1.65590171	2.23670770	0.06329992
C9	0.43875198	2.03643010	-0.55688855
N10	0.06612815	0.88752088	-1.14348228
N11	2.12183492	-0.07918603	-0.33291968
N12	3.47058044	0.95029892	1.27145998
O13	0.67904403	-1.23279401	-1.61142281

Transition state ^{3}C3
Energy (CASPT2) = -393.6783776
ZPE(CASSCF(14,10)) = 0.092251

H1	1.90357160	3.18914564	0.50404366
H2	-0.02336704	2.98165640	-1.10419248
H3	3.72566960	1.90897013	1.57461982
H4	3.78193528	0.21593434	1.55406687
H5	3.00616808	-0.67312929	-0.70611670
C6	0.82499983	-0.21118564	-0.84877427
C7	2.22922911	0.99753963	0.55198988
C8	1.58528269	2.23280478	0.11948396
C9	0.52466623	2.10991449	-0.77112415
N10	0.10448493	0.93075940	-1.21976393
N11	1.94502594	-0.14715184	0.00435568
N12	3.22883019	1.04784121	1.46983422
O13	0.48802796	-1.27683449	-1.29725171

Figure S3. Orbitals included in the active space for the calculations of the tautomers TC1 and TC2 of the 2-thiocytosine.

Tautomer TC2

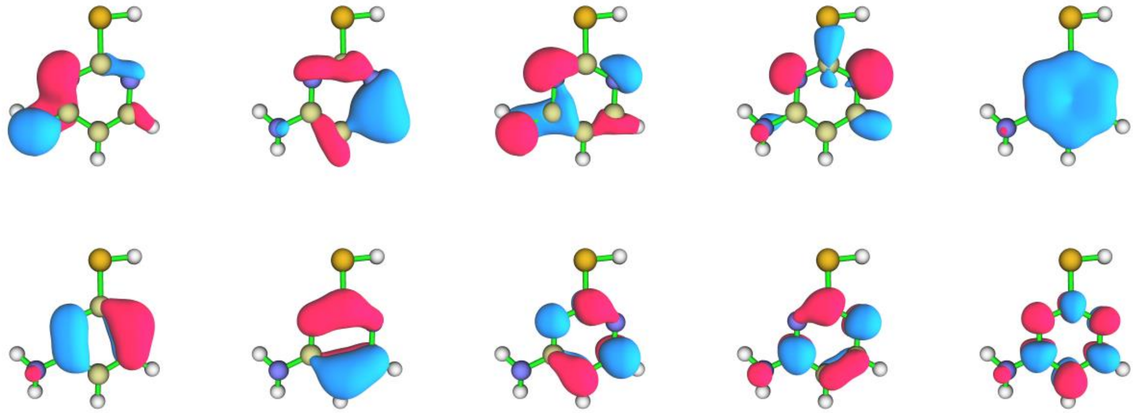

Tautomer TC1

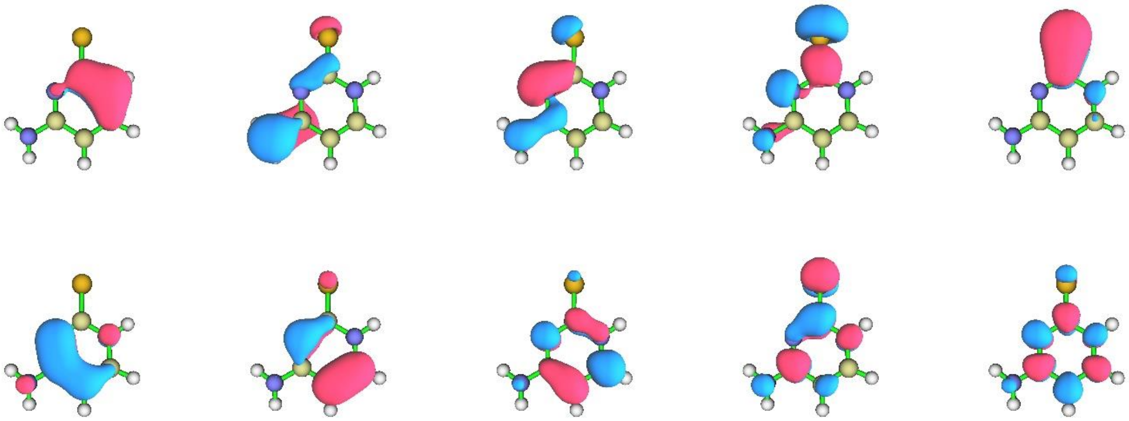

Figure S4. Active orbitals used for the recombination of the radicals TC2· + H·.

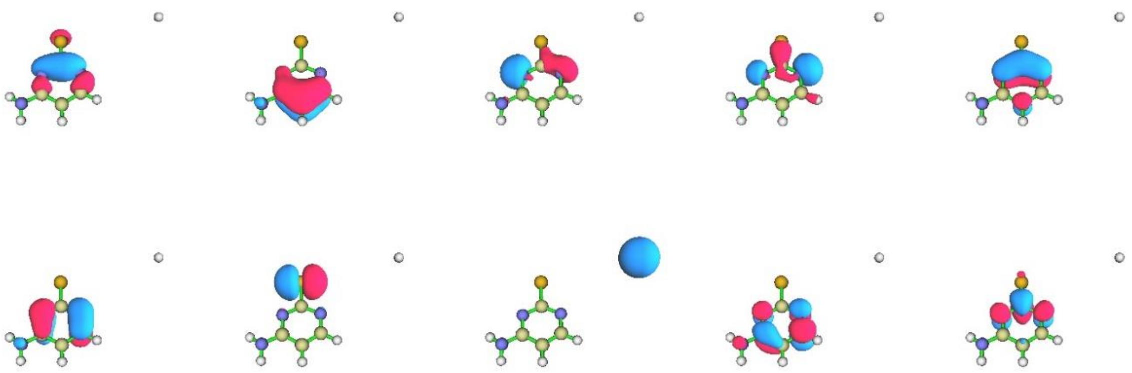

Figure S5. Orbitals included in the active space for the calculations of the tautomers C1 and C2 of the cytosine molecule.

Tautomer C2

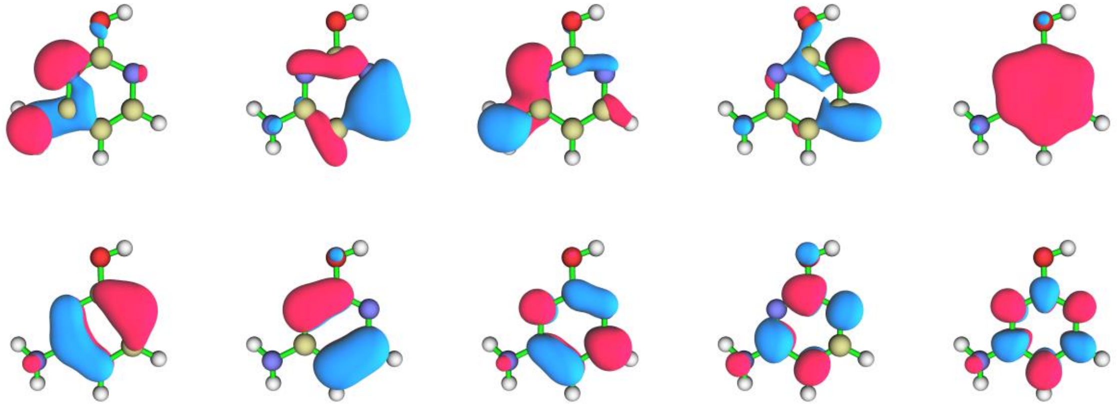

Tautomer C1

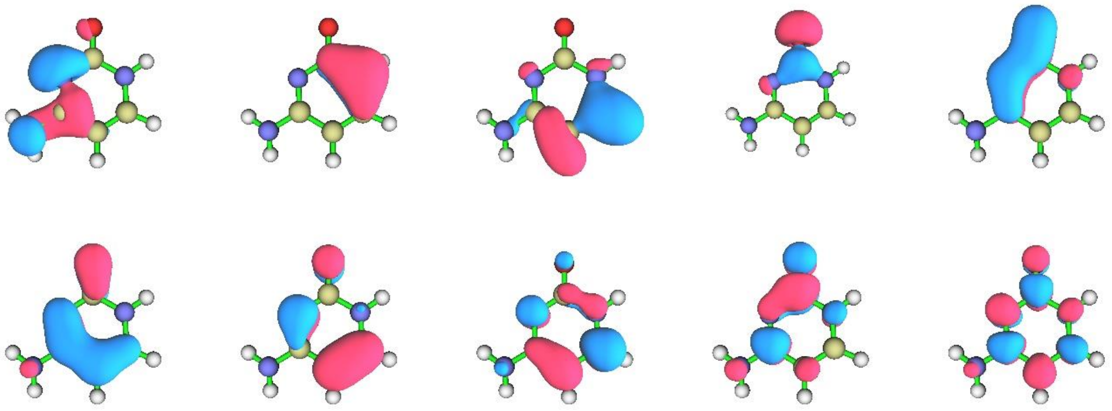

Figure S6. Active orbitals used for the recombination of the radicals C2· + H·.

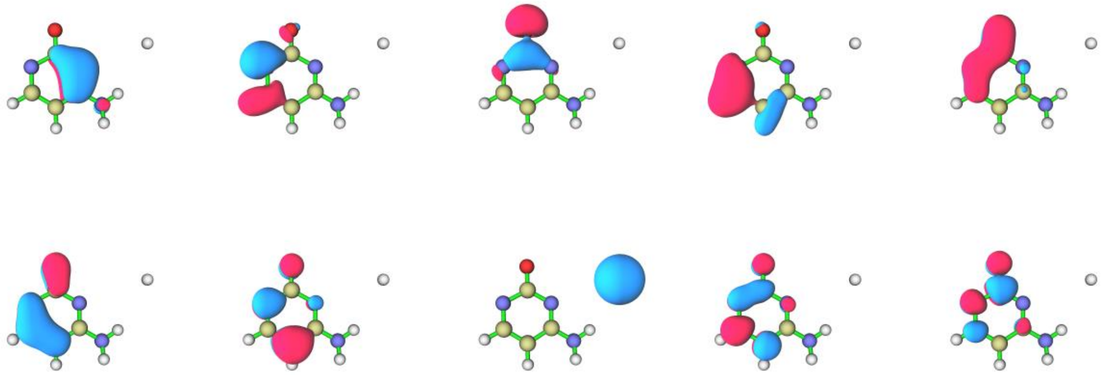

Supplement: Supplementary file 1 — ao3c09471_si_001.pdf [file ao3c09471_si_001.pdf]
